# Underestimated Cervical Extradural Hematoma Secondary to the Small Needle-Scalpel for the Treatment of Cervical Spondylosis: A Rare but Avoidable Complication

**DOI:** 10.3389/fneur.2019.00740

**Published:** 2019-07-05

**Authors:** Jinming Han, Meng-ge Yang, Qingxiang Zhang, Tao Jin

**Affiliations:** Department of Neurology, Neuroscience Center, The First Hospital of Jilin University, Changchun, China

**Keywords:** cervical extradural hematoma, small needle-scalpel, MRI, complication, cervical spondylosis

## Abstract

**Objective:** To present a case report highlighting a severe, yet avoidable, complication following small needle-scalpel treatment for cervical spondylosis.

**Introduction:** The small needle-scalpel is a miniature surgical instrument used to create intense and invasive punctures at certain acupoints with a small latch needle. It has been increasingly gaining popularity among clinicians and patients all over the world during the past years. However, severe complications after small needle-scalpel treatment have not previously been reported.

**Methods:** Here we report a 54-year-old man who recently suffered from cervical spondylosis and underwent small needle-scalpel treatment, which was performed by a rural doctor. While there were no new neurologic deficits, the patient experienced delayed functional deterioration until the onset of quadriplegia within 1 month of treatment. Magnetic resonance imaging demonstrated a C2–C7 dorsally placed extradural hematoma with severe cord compression and subcutaneous soft tissue hemorrhage.

**Results:** The patient refused urgent corrective surgery and later died due to respiratory failure.

**Conclusions:** Although small needle-scalpel therapy has many benefits, such as reducing pain, shorter expenditure, shorter period of therapy and better recovery of function, there are also many potentially severe risks, such as cervical extradural bleeding, which requires clinicians to pay more attention to avoid the complications.

## Introduction

**Figure 1:**
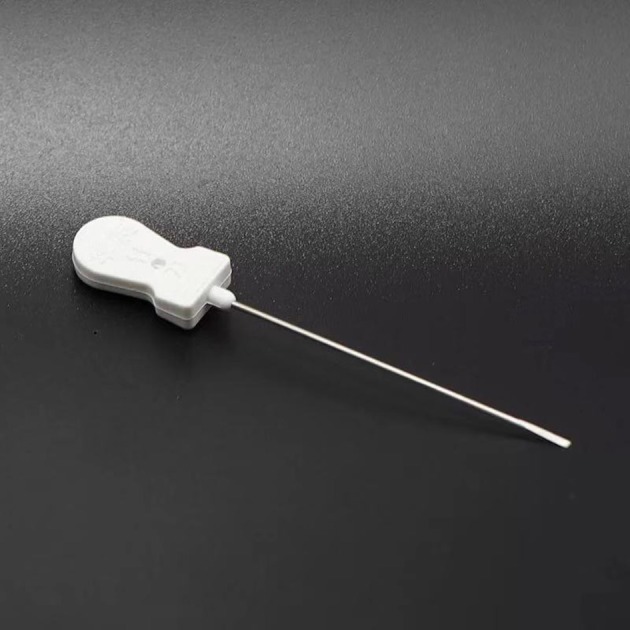
Photograph of the small needle scalpel. The shape of the small needle-scalpel looks like a needle for acupuncture with a sharp bladed tip and 0.8 mm in diameter.

**Figure 2:**
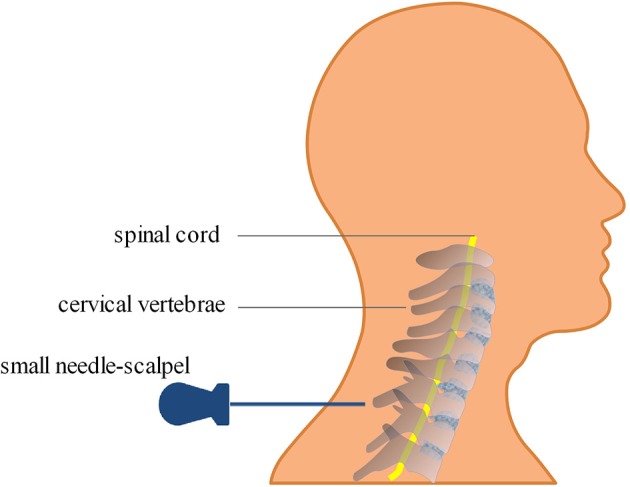
Diagram of the small needle-scalpel treatment for cervical spondylosis. The small needle-scalpel is commonly used to punctured into specific sites within the subcutaneous layer of the neck, and then scratched between the skin layer and the fascia tissue after the puncture in order to disconnect the parenchyma.

**Figure 3:**
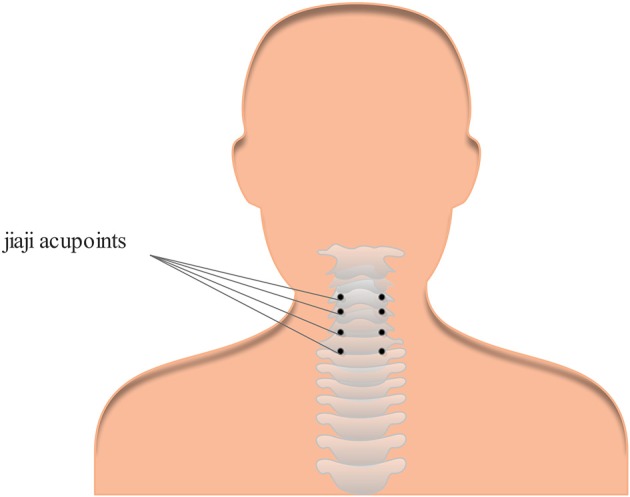
One example of the most common acupoints for cervical spondylosis. Cervical Jiaji acupoints are selected and used for the treatment of cervical spondylosis.

The small needle-scalpel is a miniature surgical instrument that creates intense and invasive punctures at certain acupoints with a small latch needle (1). The shape of the small needle-scalpel looks like a needle with a sharp bladed tip and is 0.8 mm in diameter (Figure 1). Usually, manual manipulations, such as scratching between the skin layer and the fascia tissue, are necessary after the puncture is made, mostly to disconnect the parenchyma (Figure 2). It is sometimes regarded to as a part of the concept of acupuncture, but is more invasive than normal acupuncture. It was first introduced in clinics in China in 2000 for its therapeutic effect on disorders associated with muscle pain. A recent meta-analysis demonstrated that acupuncture has a 33.41% effectiveness rate in the management of cervical spondylosis (2). It has increasingly gaining popularity among clinicians and patients throughout the world over the past years (3–6). A growing body of evidence also indicated that acupuncture may trigger a series of biological effects such as activating excitatory neurotransmission, promoting remyelination in the cuprizone-induced demyelinating animal model of multiple sclerosis, causing acupuncture-induced analgesia through purinergic signaling and reducing inflammatory response via the cholinergic anti-inflammatory pathway (7–10). Furthermore, this traditional technique has been considered an effective method for various forms of pain management including cervical spondylosis (2, 11–13). Even though effective and safe, the acupoints for the treatment of cervical spondylosis were chosen according to the theory of traditional Chinese medicine and personal clinical experience based on relevant acupoints (Figure 3), which may vary from clinician to clinician (14, 15). Here we report a previously unrecognized complication of fatal cervical extradural hematoma secondary to the small needle-scalpel for the treatment of cervical spondylosis. This case report serves to raise the awareness of potential complications followed by this kind of treatment. Thus, comprehensive assessments before and after the small needle-scalpel treatment are of importance for the patient's prognosis.

## Case Presentation

A 54-year-old Chinese man consulted a medical doctor with the complaint of recent painful neck and right upper limb without any clear causes. He had a medical history of left traumatic humeral fracture 10 years ago that did not significantly affect his recent daily life after surgery. The patient had no recent history of head or spinal trauma and did not take any related antiplatelet or anticoagulant drugs. In addition, the patient had no exposure to any toxic substances and no significant relevant family history. A clinical diagnosis of cervical spondylosis was originally considered based on X-rays of the cervical spine, and the patient received small needle-scalpel treatment for relieving pain relief, which was administered by a rural doctor. When no obvious improvement was observed 20 days later, the patient was admitted to our hospital for further diagnosis and treatment. The patient was experiencing progressive neurologic deterioration involving both upper and lower limbs. Upon neurological evaluation, he exhibited reduced response to light touch and pinprick, with a sensory level below the angle of the mandible. He had trouble during urination and significantly decreased muscle tension. He was subsequently incapable of antigravity power in his upper limbs (the left and right muscle strength was graded as 4/5 and 2/5, respectively) and had a significant loss of motor function in both lower extremities (the muscle strength was graded as 1/5). Urgent magnetic resonance imaging (MRI) of the cervical spine demonstrated a right spinal epidural hematoma located at the posterior spinal epidural space with severe cord compression and subcutaneous soft tissue hemorrhage, extending from the C2 to C7 spinal vertebral level (Figure 3). Routine laboratory investigations indicated that renal and liver functions, serum potassium and coagulation index were normal. Given his progressive symptoms and MRI results, the diagnosis of cervical extradural hematoma was finally considered. The patient had received the ventilatory and adjunctive therapy, omeprazole, mannitol, and a high dose of steroids after the diagnosis of cervical extradural hematoma. Unfortunately, the patient refused urgent surgery; thus, he continued to progressively deteriorate and later died due to respiratory failure.

## Discussion

Cervical extradural hematoma is an uncommon but highly disabling neurologic emergency in clinical practice that causes morbidity without early surgical intervention (16). The etiology of cervical extradural hematoma may be due to spontaneous and traumatic factors (16–18). Patients with cervical extradural hematoma typically present with sudden severe neck pain followed by rapidly progressive symptoms (19). Furthermore, the initial clinical signs and manifestations of cervical extradural hematoma are usually atypical, presenting unspecific clinical symptoms and mimicking other diseases that make this disease more difficult to recognize (17). However, if clinicians fail to recognize this disease early, it may lead to catastrophic consequences for patients. Here we report an underestimated, but serious case of cervical extradural hematoma that occurred secondary to small needle-scalpel for the treatment of cervical spondylosis.

**Figure 4:**
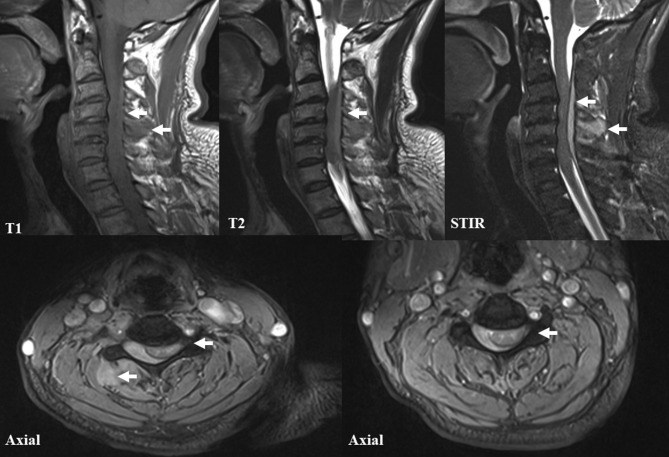
Magnetic resonance imaging (MRI) of the cervical spine. The T1-weighted, T2-weighted, short tau inversion recovery (STIR) and axial cervical MRI images demonstrated a longitudinal dorsal extradural hematoma, extending from the C2 to C7 levels. In addition, severe cord compression and related subcutaneous soft tissue hemorrhage are also noted.

Although widely considered effective and safe, several potential side effects, such as neural tissue injury, skin infection, and bleeding adhesion, have been reported with the use of acupuncture, with having a 8.6% incidence of side effects (1, 19, 20). However, secondary cervical extradural hematoma after small needle-scalpel treatment has not yet been reported. Initially diagnosed with cervical spondylosis, the patient reported here received the small needle-scalpel treatment to relieve pain while being treated by a rural medical doctor. The cause of bleeding related to the cervical extradural hematoma can be both venous and arterial in origin. Specifically, the small needle-scalpel involved the insertion of fine needles into specific sites on the body within the subcutaneous layer. Manual manipulations such as scratching between the skin layer and the fascia tissue are needed after the puncture, mostly to disconnect the parenchyma (Figure 2). In our patient, related subcutaneous soft tissue hemorrhage were obvious on MRI scans (Figure 4). In addition, this patient had no recent history of head or spinal trauma and was not taking related antiplatelet or anticoagulant drugs. Thus, the causal relationship between cervical extradural hematoma and small needle-scalpel treatment can be rated as highly probable. Small needle-scalpel treatment has the potential risk to cause fatal extradural bleeding; however, this risk is largely ignored by clinicians. In our patient, the small needle-scalpel surgery was performed by a rural medical doctor who may not have had sufficient experiences. The acupoints that were chosen for treatment vary between clinicians and inappropriate operation may damage the extradural blood vessel and cause extradural bleeding risk. In a previous study, an 47-year-old Indian patient was reported to have immediately suffered from a cervical extradural hematoma when reversed from anesthesia after spinal surgery that was noted in the posterior cervicomedullary junction and lead to a compression of the cervical cord (16). However, in our case, the patient gradually experienced progressive neurologic deterioration within 1 month after small needle-scalpel treatment without any other recent traumatic evidence. Therefore, the possibility of delayed emergence following small needle-scalpel treatment must be considered by clinical neurologists.

In conclusion, although there are many benefits, such as reducing pain, less expenditure, a shorter period of therapy, and better recovery of function, small needle-scalpel treatment may also have the potential to cause severe risks, such as cervical extradural bleeding which requires clinicians to pay more attention to avoid related complications. Precisely choosing the acupoints, standardized surgery and long-term follow-up may avoid related complications followed by small needle-scalpel treatment.

## Ethics Statement

As this is a case report without experimental intervention, no formal research ethics approval is required. The relatives of this patient provided written informed consent to the publication of the information and images related to this case report.

## Author Contributions

JH drafted the manuscript. MY and QZ helped to prepare the figures and collected data. TJ performed a critical revision.

### Conflict of Interest Statement

The authors declare that the research was conducted in the absence of any commercial or financial relationships that could be construed as a potential conflict of interest.
